# Cutaneous Small Vessel Vasculitis Secondary to Clozapine Use in a 22-Year-Old Man With Treatment-Resistant Schizophrenia

**DOI:** 10.7759/cureus.106430

**Published:** 2026-04-04

**Authors:** Kumaran O Ravindran, Vivian Kapil V, Krishnaswamy Madhavan, Ravindran O Somasundaram

**Affiliations:** 1 Institute of Internal Medicine, Madras Medical College, Chennai, IND; 2 Psychiatry, Panimalar Medical College Hospital and Research Institute, Chennai, IND; 3 General Medicine, SRM Medical College Hospital and Research Institute, Chengalpattu, IND; 4 Clinical Psychology, Sri Ramachandra Institute of Higher Education and Research, Chennai, IND

**Keywords:** antipsychotic adverse effects, clozapine, cutaneous small vessel vasculitis, drug-induced vasculitis, leukocytoclastic vasculitis, treatment-resistant schizophrenia

## Abstract

Clozapine is the most effective drug for treatment-resistant schizophrenia (TRS) but is associated with several adverse effects, including rare immune-mediated reactions. Cutaneous small vessel vasculitis (CSVV), also known as leukocytoclastic vasculitis, is an uncommon complication that typically presents as palpable purpura involving dependent areas of the body.

We describe a case of a 22-year-old male patient with TRS who developed a painful, non-blanching purpuric, papular rash over both lower limbs two weeks after initiation and dose escalation of clozapine. Laboratory evaluation showed mild leukocytosis with eosinophilia, while renal, hepatic, infectious, and immunological investigations were normal, and there was no evidence of systemic involvement. In view of the clear temporal association with clozapine initiation and absence of alternative causes, a diagnosis of drug-induced CSVV was made.

Clozapine was discontinued, and the patient was transitioned to quetiapine for ongoing psychiatric management. The vasculitic lesions were managed with systemic corticosteroids, antihistamines, pentoxifylline, and supportive measures, resulting in marked improvement within two weeks and complete resolution over six weeks without recurrence. Causality assessment using the Naranjo adverse drug reaction probability scale gave a score of 6, suggestive of a probable association with clozapine. Rechallenge was avoided due to the risk of recurrence.

This case emphasizes the need for clinicians to recognize rare immune-mediated cutaneous reactions associated with clozapine. Early recognition and prompt discontinuation of the offending agent are essential to prevent progression and ensure favorable outcomes.

## Introduction

Clozapine is a second-generation antipsychotic and remains the most effective pharmacological treatment for patients with treatment-resistant schizophrenia (TRS), defined as an inadequate response to at least two antipsychotics of adequate dose and duration [1,2]. Despite its superior efficacy, clozapine is associated with a wide spectrum of adverse effects ranging from common dose-related effects to rare but potentially serious immune-mediated reactions [3]. Among these, cutaneous small vessel vasculitis (CSVV), also referred to histologically as leukocytoclastic vasculitis (LCV), is an uncommon but clinically significant complication [4,5]. Recognizing this uncommon but potentially serious adverse effect is important, as early identification can prevent complications such as renal involvement and systemic vasculitis and ensure appropriate management. CSVV is characterized by inflammation of post-capillary venules and typically presents as palpable purpura, predominantly involving dependent areas of the body [5-7]. It is most commonly associated with infections, medications, autoimmune disorders, and malignancies, although many cases remain idiopathic. We report a case of clozapine-induced CSVV in a young man with TRS, highlighting diagnostic challenges, management, and implications for clinical monitoring.

## Case presentation

Patient profile

A 22-year-old male patient with a diagnosis of TRS was initiated on clozapine following poor response to adequate trials of risperidone and olanzapine. He had no prior medical comorbidities, no history of autoimmune disease, infections, or malignancy, and no known drug allergies.

Clinical course

**Figure 1:**
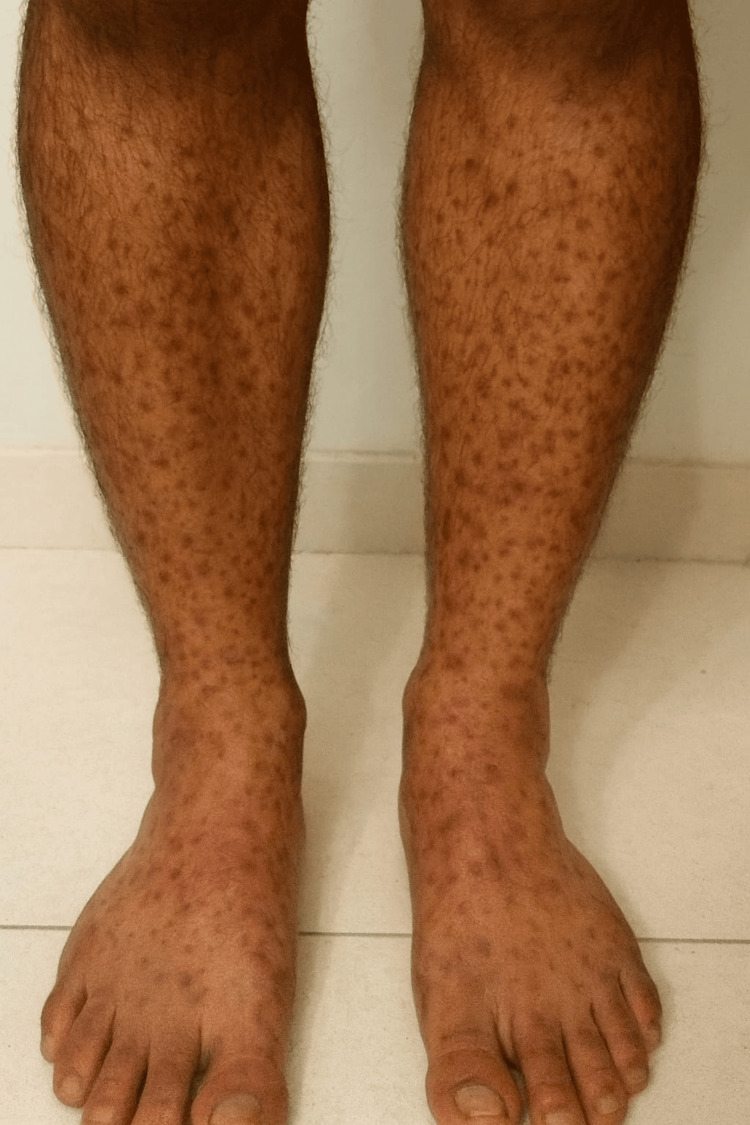
Cutaneous small vessel vasculitis involving bilateral lower limbs Clinical photograph demonstrating multiple, round-to-oval palpable purpuric lesions measuring 1-3mm, symmetrically distributed over the lower legs and feet.

Clozapine was initiated at 12.5 mg/day and gradually titrated to 100 mg/day over two weeks, with a planned target dose of 200 mg/day at the end of four weeks. Two weeks after initiation, the patient developed a painful, non-blanching, purpuric, papular rash predominantly over both lower limbs (Figure 1). The lesions were associated with mild pruritus and peripheral edema. There were no systemic symptoms such as fever, arthralgia, abdominal pain, diarrhea, cough, dyspnea, hematuria, or frothy urine. The rash and associated discomfort interfered with his ability to stand for long periods and increased his distress. Dermatological examination revealed multiple, round-to-oval palpable purpuric lesions measuring 1-3 mm, symmetrically distributed over the lower legs and feet. The patient's vital parameters at presentation are shown in Table 1. 

**Table 1:** Patient vital parameters at presentation

Parameter	Value	Reference Range	Interpretation
Temperature	99°F	97–99°F	Afebrile
Heart Rate	88 bpm	60–100 bpm	Normal
Blood Pressure	112/72 mmHg	90/60–120/80 mmHg	Normal
Respiratory Rate	18/min	12–20/min	Normal
Oxygen Saturation	98% (RA)	≥95%	Normal

Investigations

A summary of laboratory investigations performed at presentation is shown in Table 2.

**Table 2:** Laboratory investigations

Investigation	Result	Reference Range	Interpretation
Full blood count			
White blood cells (×10⁹/L)	11.8	4.5–11	Mild leukocytosis consistent with inflammatory/drug reaction
Hemoglobin (g/dL)	13.2	13–17	No anemia
Platelets (×10⁹/L)	268	150–400	Thrombocytopenia excluded as the cause of purpura
Absolute neutrophil count (cells/µL)	8,400	2,000–7,000	Mild neutrophilia consistent with inflammatory response
Absolute eosinophil count (cells/µL)	650	<500	Consistent with drug-induced hypersensitivity reaction
Liver biochemistry			
Aspartate transaminase (U/L)	32	<40	Normal
Alanine transaminase (U/L)	28	<40	Normal
Serum albumin (g/L)	41	35–50	No systemic inflammatory or protein-losing state
Alkaline phosphatase (U/L)	96	<120	Normal
Gamma-glutamyl transferase (U/L)	44	5–40	Mild elevation without clinical significance
INR	1.0	<1.1	Normal coagulation; purpura not due to coagulopathy
Renal profile			
Serum creatinine (mg/dL)	0.9	0.6–1.2	Normal
Blood urea (mg/dL)	18	6–24	Normal
Urinalysis	Normal	—	No hematuria or proteinuria suggesting renal vasculitis
Urine microscopy	No RBC casts or active sediment	—	Glomerular involvement unlikely
Electrolytes			
Serum sodium (mmol/L)	138	135–145	Normal
Serum potassium (mmol/L)	4.3	3.5–5.5	Normal
Serum calcium (mmol/L)	2.4	2.2–2.7	Normal
Serum magnesium (mg/dL)	1.9	1.6–2.5	Normal
Thyroid function			
Thyroid-stimulating hormone (mIU/L)	2.1	0.5–5.0	Thyroid dysfunction excluded
Inflammatory markers			
Erythrocyte sedimentation rate (mm/hr)	28	<20	Mild inflammatory activity consistent with cutaneous vasculitis
C-reactive protein (mg/L)	3	<5	No significant systemic inflammation
Immunological tests			
Antinuclear antibody	Negative	Negative	Connective tissue disease unlikely
p-ANCA	Negative	Negative	ANCA-associated vasculitis unlikely
Rheumatoid factor	Negative	Negative	Autoimmune vasculitis unlikely
Complement C3 (mg/dL)	110	90–180	No complement consumption
Complement C4 (mg/dL)	28	10–40	No complement consumption
Infectious screening			
Hepatitis B surface antigen	Negative	Negative	Infection-related vasculitis unlikely
Hepatitis C antibody	Negative	Negative	Infection-related vasculitis unlikely
HIV serology	Negative	Negative	Secondary vasculitis unlikely
Anti-streptolysin O titer (IU/mL)	82	<200	Recent streptococcal infection unlikely

**Figure 2:**
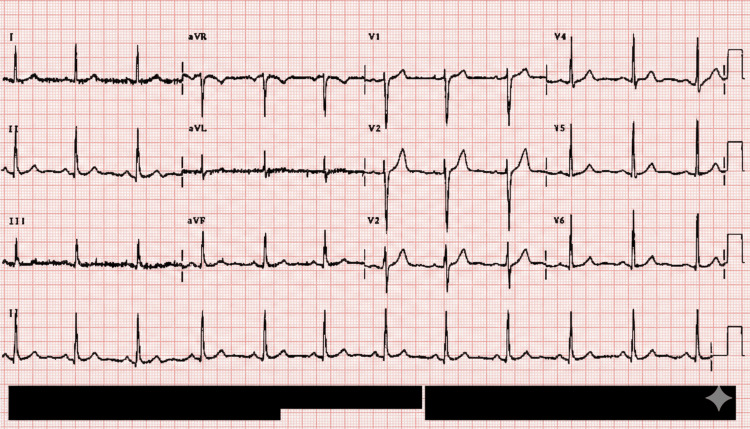
Twelve-lead electrocardiogram showing normal sinus rhythm with normal axis and intervals. No significant ST-T changes or conduction abnormalities are observed

**Figure 3:**
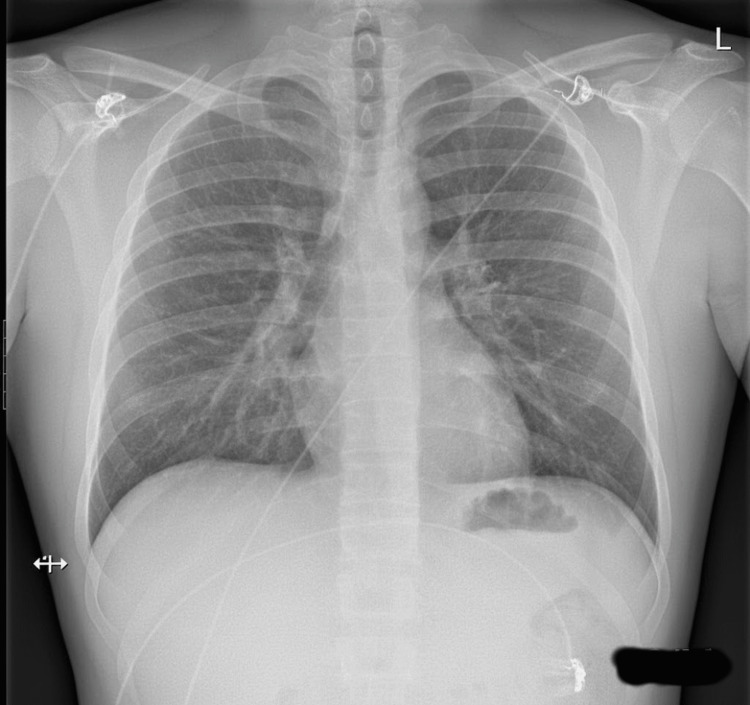
Frontal chest radiograph demonstrating normal cardiopulmonary findings. There is no evidence of pulmonary infiltrates, nodules, hemorrhage, pleural effusion, or mediastinal widening. The cardiac silhouette is normal in size. These findings suggest absence of radiographic pulmonary involvement

Given the absence of systemic involvement, normal urine examination, and clear temporal association with clozapine initiation, a skin biopsy was deferred. The clinical picture was consistent with drug-induced CSVV, with clozapine identified as the most likely offending agent [8,9]. Electrocardiography revealed a normal sinus rhythm without evidence of conduction defects or ischemic changes (Figure 2). Chest radiography showed normal cardiopulmonary findings with no evidence of infiltrates, effusion, or other abnormalities (Figure 3).

Diagnosis

The differential diagnoses (Table 3) considered included IgA vasculitis, antineutrophil cytoplasmic antibody (ANCA)-associated vasculitis, cryoglobulinemic vasculitis, connective tissue disease-associated vasculitis, malignancy-associated vasculitis, thrombocytopenic purpura, and drug-related exanthematous eruptions [5,6].

**Table 3:** Differential diagnoses considered in the evaluation of cutaneous small vessel vasculitis ANCA: Antineutrophil cytoplasmic antibody

Differential Diagnosis	Reason for Consideration	Reason for Exclusion
IgA vasculitis (Henoch–Schönlein purpura)	Palpable purpura with leukocytoclastic vasculitis can mimic cutaneous small vessel vasculitis.	No abdominal pain, arthritis, or renal involvement; urinalysis normal.
ANCA-associated vasculitis	Systemic small-vessel vasculitis may present with cutaneous purpura.	ANCA negative; no renal, pulmonary, or neurologic involvement.
Cryoglobulinemic vasculitis	Immune-complex vasculitis causing palpable purpura of the lower limbs.	Cryoglobulin test negative; no hypocomplementemia or associated infection such as hepatitis C.
Connective tissue disease–associated vasculitis (e.g., systemic lupus erythematosus, rheumatoid arthritis)	Autoimmune diseases can cause secondary small-vessel vasculitis.	Autoimmune serology negative; no clinical features of systemic autoimmune disease.
Malignancy-associated vasculitis	Paraneoplastic vasculitis may present with leukocytoclastic purpura.	No constitutional symptoms or investigations suggesting malignancy.
Thrombocytopenic purpura	Purpuric lesions from platelet disorders can resemble vasculitis.	Platelet count normal; lesions palpable rather than non-palpable.
Drug-related exanthematous eruption	Drug eruptions may occur temporally after medication exposure.	Non-blanching palpable purpura with biopsy-proven leukocytoclastic vasculitis.

Normal platelet count, negative immunological markers, absence of renal or gastrointestinal involvement, and spontaneous improvement following drug withdrawal supported the diagnosis of clozapine-induced CSVV.

Management

Clozapine was immediately discontinued. The patient was switched to quetiapine, titrated to 600 mg/day, which resulted in partial control of psychotic symptoms. For management of CSVV, oral prednisolone was initiated at 0.5 mg/kg/day in three divided doses. Adjunctive therapy included cetirizine 10 mg once daily and pentoxifylline 1200 mg/day in three divided doses. The patient was advised to rest and elevate the lower limbs.

Marked clinical improvement was observed within two weeks, with resolution of purpura and edema. Corticosteroids were tapered gradually over four weeks without recurrence of lesions. Complete resolution occurred within six weeks of clozapine discontinuation. A repeat complete blood count performed eight weeks later was normal. The patient was reviewed after six months and found to have no new rash. In view of the probable causal link and the availability of alternative antipsychotic strategies, the treating team and family agreed not to pursue clozapine rechallenge.

## Discussion

Association between clozapine and vasculitis

Drug-induced vasculitis accounts for a significant proportion of cases of CSVV [10,11]. Clozapine, although infrequently implicated, has been associated with immune-mediated hypersensitivity reactions, including myocarditis, hepatitis, and vasculitis [3,12]. Few cases of clozapine-associated leukocytoclastic vasculitis have been reported in the literature. Mukherjee et al. reported a case of leukocytoclastic vasculitis secondary to clozapine, with onset shortly after drug initiation and complete resolution following withdrawal, closely mirroring the time course and clinical features observed in our patient [13]. In addition, Fujimoto et al. described clozapine-induced ANCA-associated vasculitis with leukocytoclastic vasculitis, providing further evidence for an immune-mediated mechanism underlying clozapine-associated vasculitic reactions [14]. These reports support the plausibility of clozapine as a trigger for immune-complex-mediated and antibody-driven vasculitis. The temporal relationship between drug exposure and symptom onset, the absence of alternative etiologies, and the resolution after withdrawal strongly support a causal association in this case.

Pathophysiology

The proposed mechanism of clozapine-induced CSVV is a type III (immune complex-mediated) hypersensitivity reaction [5,11]. Clozapine or its reactive metabolites may act as haptens, forming neoantigens that elicit IgG or IgM antibody production. Circulating immune complexes deposit in post-capillary venules, activate the classical complement pathway (C3a, C5a), and recruit neutrophils. Subsequent release of proteolytic enzymes and reactive oxygen species leads to endothelial injury, fibrinoid necrosis, leukocytoclasia, and erythrocyte extravasation. The presence of eosinophilia, as seen in this patient, further supports a drug-induced etiology [5].

Diagnostic considerations

While skin biopsy remains the gold standard for confirming leukocytoclastic vasculitis [5,6], it may be deferred in clinically straightforward cases with isolated cutaneous involvement and a clear drug trigger [8]. The 2012 Chapel Hill Consensus Conference classifies such cases under single-organ vasculitis limited to the skin [6].

Treatment considerations

Immediate withdrawal of the offending drug is the cornerstone of management [7,11]. Systemic corticosteroids are effective in controlling inflammation in moderate to severe cases, while adjunctive agents such as antihistamines and pentoxifylline may provide symptomatic benefit [7-9]. The prognosis of drug-induced CSVV is generally favorable, with most cases resolving completely after drug discontinuation [11]. The possibility of cross-reactivity with other structurally related antipsychotics, particularly olanzapine and quetiapine, should be considered when selecting alternative therapy following clozapine discontinuation. Careful drug selection and close monitoring are warranted to minimize the risk of recurrence of hypersensitivity reactions [14].

Causality assessment

Application of the Naranjo adverse drug reaction probability scale yielded a score of 6, indicating a probable adverse drug reaction [15]. In view of the probable causal link and the availability of alternative antipsychotic strategies, the treating team and family agreed not to pursue clozapine rechallenge. 

Implications for clozapine monitoring

This case underscores the importance of maintaining vigilance for rare immune-mediated adverse effects of clozapine in addition to routine monitoring for hematological and cardiac complications [3]. Early recognition of cutaneous vasculitis and prompt drug withdrawal can prevent progression to systemic involvement and reduce morbidity.

## Conclusions

Clozapine-induced CSVV is a rare but clinically important adverse effect. This case highlights the need for a high index of clinical suspicion when new-onset palpable purpura develops in patients receiving clozapine. Early diagnosis, exclusion of systemic disease, and prompt discontinuation of the offending agent result in excellent outcomes.
